# Comparison and contrast of genes and biological pathways responding to Marek’s disease virus infection using allele-specific expression and differential expression in broiler and layer chickens

**DOI:** 10.1186/1471-2164-14-64

**Published:** 2013-01-30

**Authors:** Sudeep Perumbakkam, William M Muir, Alexis Black-Pyrkosz, Ron Okimoto, Hans H Cheng

**Affiliations:** 1 Department of Animal Science, Purdue University, West Lafayette, IN, 47907, USA; 2 Avian Diseases and Oncology Laboratory, USDA, ARS, East Lansing, MI, 48823, USA; 3 Cobb-Vantress, Siloam Springs, AR, 72761, USA

**Keywords:** Marek’s disease, Genetic resistance, RNA-Seq, Allele-specific expression, Biological pathways

## Abstract

**Background:**

Marek’s disease (MD) is a commercially important neoplastic disease of chickens caused by the Marek’s disease virus (MDV), a naturally occurring oncogenic alphaherpesvirus. Enhancing MD genetic resistance is desirable to augment current vaccines and other MD control measures. High throughput sequencing was used to profile splenic transcriptomes from individual F_1_ progeny infected with MDV at 4 days of age from both outbred broilers (meat-type) and inbred layer (egg-type) chicken lines that differed in MD genetic resistance. The resulting information was used to identify SNPs, genes, and biological pathways exhibiting allele-specific expression (ASE) in response to MDV infection in each type of chicken. In addition, we compared and contrasted the results of pathway analyses (ASE and differential expression (DE)) between chicken types to help inform on the biological response to MDV infection.

**Results:**

With 7 individuals per line and treatment group providing high power, we identified 6,132 single nucleotide polymorphisms (SNPs) in 4,768 genes and 4,528 SNPs in 3,718 genes in broilers and layers, respectively, that exhibited ASE in response to MDV infection. Furthermore, 548 and 434 genes in broilers and layers, respectively, were found to show DE following MDV infection. Comparing the datasets, only 72 SNPs and 850 genes for ASE and 20 genes for DE were common between the two bird types. Although the chicken types used in this study were genetically different, at the pathway level, both TLR receptor and JAK/STAT signaling pathways were enriched as well as exhibiting a high proportion of ASE genes, especially at the beginning of both above mentioned regulatory pathways.

**Conclusions:**

RNA sequencing with adequate biological replicates is a powerful approach to identify high confidence SNPs, genes, and pathways that are associated with transcriptional response to MDV infection. In addition, the SNPs exhibiting ASE in response to MDV infection provide a strong foundation for determining the extent to which variation in expression influences MD incidence plus yield genetic markers for genomic selection. However, given the paucity of overlap among ASE SNP sets (broilers vs. layers), it is likely that separate screens need to be incorporated for each population. Finally, comparison of gene lists obtained between these two diverse chicken types indicate the TLR and JAK/STAT signaling are conserved when responding to MDV infection and may be altered by selection of genes exhibiting ASE found at the start of each pathway.

## Background

Marek’s disease (MD) is a lymphoproliferative disease of chickens caused by the Marek’s disease virus (MDV or *Gallid herpesvirus 2*), a naturally occurring alphaherpesvirus [1,2]. The virus targets lymphoid tissue such as the bursa of Fabricius, thymus, and spleen, where it infects B and T cells [3]. The pathological characteristics of MD include mononuclear infiltration of the peripheral nerves, gonads, iris, various viscera, muscles, and skin. Susceptible chickens develop lymphomas in visceral tissues and enlarged nerves resulting in paralysis, blindness, and eventually death. Economic losses to the poultry industry due to condemnation in broilers (meat-type) and reduction in egg production in layers (egg-type) are estimated to be $1-2 billion per year [4]. Due to the persistent nature of the virus in the feather dander shed from MDV-infected birds, all commercial chickens are exposed at a very early age. Although MD vaccines can prevent the formation of tumors, they do not prevent viral replication and bird-to-bird spread. The lack of sterilizing immunity is thought to be a major contributing factor for MDV field strains evolving to higher virulence [5], which can result in unpredictable and devastating disease outbreaks in vaccinated commercial flocks.

An attractive solution to augment MD vaccinal and husbandry control measures is to increase genetic resistance in commercial chicken lines through marker-assisted or genomic selection, which avoids the need to expose elite flocks under selection to a hazardous pathogen. In the past, focusing on the experimental White Leghorn (layer) lines 6_3_ and 7_2_, which are MD resistant and susceptible, respectively, we have utilized multiple techniques such as genome-wide QTL scans [6,7], transcript profiling with microarrays [8], and protein-protein interaction [9] screens to understand the response to MDV infection and MD genetic resistance. Integrating the results from these approaches identified three genes and many other strong candidates that confer genetic resistance to MD [8,10,11]. While promising, these genes account for only a small fraction of the observed genetic variation, a situation that is similar for many other complex traits.

Allele-specific expression (ASE) is a powerful technique that measures the expression of each allele via a marker single nucleotide polymorphism (SNP) within a RNA sample. When a gene shows allelic imbalance, it is sufficient to identify a *cis*-acting regulatory element [12]. The key advantage of this approach is identification of a SNP exhibiting ASE, though likely not to be causative, is in tight linkage disequilibrium with the causative polymorphism, thus, essentially identifying a high-confidence candidate gene (genetic factor) with expression variation. And since variation in gene expression is thought to be a major factor accounting for phenotypic variation, genes with ASE SNPs provide candidates and markers that may account for the complex trait of interest.

In this study, the primary aim was to use ASE to identify SNPs associated with variation in transcriptional response to MDV infection in both broilers and layers, which would provide a strong foundation for future evaluations of genetic resistance to MD. In addition, the resulting information allowed us to compare the identified SNPs, genes, and pathways enriched between these two diverse chicken types. Furthermore, since RNA sequencing (RNA-Seq) data is comparable to microarray data, gene counts were used to identify genes differentially expressed (DE) in response to MDV infection and, subsequently, used to evaluate the biological pathways that were shared between the broiler and layer chickens.

## Results

### MD incidence in broilers

As the long-term objective is to improve MD genetic resistance through genomic selection, we first needed to identify broiler pure lines that differed substantially in MD incidence; layer lines 6_3_ and 7_2_ are already known to be MD resistant and susceptible, respectively [13]. Anecdotal information suggested that Cobb-Vantress lines “Red” and “Blue” used in this study differed in MD genetic resistance. We confirmed these differences in two MDV challenge trials (Table 1), where the Red line birds had about twice the MD incidence as those from the Blue line.

**Table 1:** Marek’s disease (MD) incidence in two pure line broilers

**Pure line**	**Trial 1**	**Trial 2**
Red	57%	58%
Blue	32%	30%

### Whole-Genome transcriptomics and ASE SNP discovery

In the present experiment, we sequenced RNA samples from 7 uninfected and 7 infected birds for each type (broiler or layer). For the broiler dataset, with one exception, the average number of single end raw reads was approximately 27 million (Additional file 1: Table S1), and after quality trimming was between 21 million and 29 million. Tophat/Bowtie [14] aligned 68-81% of the reads to the chicken genome with nearly 98.5% of the reads in the resulting alignments having a mapping quality score of 30 or higher (MAPQ≥30). These high quality scores were necessary to confidently call SNPs with a minimum of false positives. Using Freebayes [15], SNPs were called within alignments from similar treatment samples (e.g., all 7 MDV-infected layers), with criteria to contain at least 14 reads in the population. The average number of SNPs finally called was in approximate range of 200,000-310,000 per sample in the broilers.

For layer samples, similar quality control measures were undertaken to reduce false positives SNPs. For these RNAs, sequencing was performed using the Illumina HiSeq platform, which not only produced more reads compared to the broiler-generated data (Illumina GAII) but also produced longer pair end fragments (Additional file 1: Table S2). The average number of reads ranged from 40 to 95 million per sample. The average number of SNPs called was in approximate range of 195,000-390,000 per sample in the layers. The increase in reads provided more confidence in SNP calls (read depth) but did not significantly increase the number of SNPs called between the samples (broilers vs. layers). Also, higher read depth did not significantly change the Tophat/Bowtie [14] alignment statistics between the samples (broilers vs. layers).

An analysis of variance (ANOVA) was used to identify the SNPs that showed ASE due to MDV infection in broilers or layers. After further filtering of the raw SNP data, an ANOVA was performed on 150,403 SNPs from the broiler SNP dataset and 134,953 SNPs for the layer that passed our test criteria (see Materials and Methods). Based on ANOVA significance (p<0.05), the number of SNPs exhibiting ASE in response to MDV infection in broilers and layers was 6,132 and 4,528, respectively (Additional file 1: Tables S3 and S4). No correction was made for multiple testing because the list of genes would be tested subsequently for pathway enrichment. In the Fisher exact test invoked in DAVID [16] to identify enriched pathways, the proportion of genes submitted must be significantly greater than the proportion of genes found by chance. In this way, Type 2 errors (false negatives) are reduced in the first step while the pathway test in the second step includes an experiment wise error rate to control for Type 1 errors (false positives).

### Classification of ASE SNPs

The significant SNPs exhibiting ASE in response to MDV infection for both broilers and layers were distributed on chromosomes 1 to 28 of the chicken genome, with the number of SNPs per each chromosome roughly proportional to the size of the each chromosome in the chicken genome assembly (Additional file 2: Figure S1).

To locate these SNPs on the chicken genome with respect to genes and classify them based on function, ANNOVAR [17] was used. In the broiler population, the largest number of SNPs (1,780 or 29.0%) was classified as exonic (Table 2). The second largest number of SNPs fell in the intergenic group (1,610 or 26.3%), and the third largest group were located downstream of a gene (1,073 or 17.5% of the SNPs). As RNA was sequenced and analyzed, SNPs in both of these latter categories reflect the incomplete or inaccurate annotation of the chicken genome. Similarly in layers, the two largest classifications of SNPs associated with intergenic (1,059 or 25.4%) and exonic (1,501 or 24.8%) regions (Table 2).

**Table 2:** Classification of allele-specific expression (ASE) SNPs responding to Marek’s disease virus (MDV) infection in broilers and layers

**SNP type**	**Broiler**		**Layer**	
	**SNPs**	**Genes**	**SNPs**	**Genes**
Downstream	1,073	875	583	524
Exonic	1,780	1,501	1,123	947
Exonic Splicing	39	38	41	41
Intergenic	1,610	1,059	1,150	868
Intronic	541	394	913	691
ncRNA_exonic	1	1	3	3
Splicing	8	8	7	7
Upstream	94	88	118	109
Upstream:Downstream	47	41	41	36
UTR’3	863	694	467	414
UTR’5	74	67	81	77
UTR’3: UTR’5	2	2	1	1
Total	6,132	4,768	4,528	3718

A nucleotide position match between broiler and layer ASE SNPs showed 72 SNPs matched at the exact position (Additional file 1: Table S5). These 72 SNPs were distributed in 71 genes (Table 3). At the gene level, there were 850 genes showing ASE in response to infection that were common between the two chicken types (Table 3). The observed overlap was not significant suggesting different genes are responsible for conferring MD resistance between the broiler and layer lines examined.

**Table 3:** Classification of allele-specific expression (ASE) SNPs in response to Marek’s disease virus (MDV) infection in common between broilers and layers at the nucleotide position and gene level

**SNP type**	**Nucleotide-level match**		**Gene-level match**
	**SNPs**	**Genes**	**Genes**
Downstream	12	11	86
Exonic	18	18	231
Exonic Splicing	0	0	0
Intergenic	25	25	301
Intronic	6	6	117
ncRNA_exonic	0	0	0
Splicing	0	0	0
Upstream	1	1	9
Upstream:Downstream	0	0	2
UTR’3	9	9	101
UTR’5	1	1	3
UTR’3: UTR’5	0	0	0
Total	72	71	850

The ASE data were further analyzed to quantify the biological implications due to the presence of a SNP. A function-based approach using ANNOVAR classified the SNPs as synonymous, nonsynonymous, stop gain, and stop loss (Table 4). In broiler and layers, 1,818 and 1,163 SNPs, respectively, were identified (Additional file 1: Tables S6 and S7). The largest category in both chicken types was synonymous implying that the SNPs were neutral and downstream amino acid changes were not significant. The second largest category in both the chicken breeds was nonsynonymous SNPs. A very small number of the SNPs fell into the other two categories (stop gains and stop loss).

**Table 4:** Functional classification of allele-specific expression (ASE) SNPs in response to Marek’s disease virus infection from broilers and layers in known genes

**SNP type**	**Broiler**			**Layer**			**Common**
	**SNPs**	**% of SNPs**	**Gene**	**SNPs**	**% of SNPs**	**Gene**	**Gene**
Nonsynonymous	442	24.3	399	317	27.3	291	14
Stop gain	4	0.2	4	5	0.4	5	0
Stop loss	2	0.1	2	1	0.1	1	0
Synonymous	1,370	75.4	936	840	72.2	735	162
Total	1,818	100	1,341	1,163	100	1,032	176

### Differential expression (DE) analysis in response to MDV infection

DE was estimated by obtaining count data from each biological sample (broilers and layers) using the Htseq script [18]. The ENSEMBL exon annotation file [19] identified 17,934 chicken genes of which 15,261 and 15,468 genes were expressed in the broilers and layers, respectively. DESeq analysis [20] was used to estimate DE and a total of 548 and 434 genes were revealed following MDV infection (p<0.05) in broilers and layers, respectively (Additional file 1: Tables S8 and S9). The fold change ranged from −6.41 to 35.0 and −8.86 to 48.0 in broilers and layers, respectively. Of the genes that were significant, only 20 were common between the broiler and layer chickens.

### Pathway analysis of ASE and DE genes

To identify potential common pathways, gene lists obtained by ASE and DE analysis were analyzed for biological process and pathway enrichment using DAVID [16]. Due to the likely limited effect of SNPs falling in the intergenic, intronic, and downstream regions of the chicken genome (based on ANNOVAR classification), we further filtered the input gene list to only include genes that corresponded to SNPs that were found to be synonymous, nonsynonymous, or located at the 5’ UTR, and 3’ UTR regions. From the initial gene list (Table 2), 2,156 and 1,207 genes following ASE analysis in the broiler and layer samples, respectively, were queried. Using functional annotation clustering, at the highest classification stringency, 109 and 63 clusters were formed in broilers and layers, respectively. When an enrichment cutoff at >1.0 was used, only 12 and 6 clusters were chosen in broilers and layer samples, respectively (Additional file 1: Table S10 and S11). There were no common enriched clusters between both datasets. The pathways analysis invoked in DAVID yielded 13 and 6 pathways in the broiler and layer samples, respectively (Table 5). Of these samples, there were three pathways that were common between the two bird types: apoptosis, DNA replication, and amino and nucleotide sugar metabolism. There were additional pathways found that were unique to each chicken type (Table 5). DAVID analysis of nonsynonymous ASE SNPs from broilers and layers also resulted in no common pathways that were found to be enriched (Additional file 1: Tables S12 and S13) but 14 genes were obtained that were common to both bird lines (Additional file 1: Table S14). Furthermore, out of the 14 common genes obtained, 6 genes were functional annotated using DAVID (Additional file 1: Table S15).

**Table 5:** DAVID analysis of allele-specific expression (ASE) genes in response to Marek’s disease virus (MDV) infection (P<0.05) in broilers and layers

**Category**	**Term**	**Count**	**%**	**P-Value**
A. Broilers				
KEGG_PATHWAY	Nucleotide excision repair	13	0.6	2.30E-03
KEGG_PATHWAY	DNA replication	10	0.5	1.70E-02
KEGG_PATHWAY	Fatty acid metabolism	10	0.5	2.90E-02
KEGG_PATHWAY	Apoptosis	19	0.9	3.10E-02
KEGG_PATHWAY	Ribosome	19	0.9	3.10E-02
KEGG_PATHWAY	Lysosome	23	1.1	3.50E-02
KEGG_PATHWAY	Basal transcription factors	9	0.4	3.90E-02
KEGG_PATHWAY	p53 signaling pathway	15	0.7	5.50E-02
KEGG_PATHWAY	Base excision repair	8	0.4	6.60E-02
KEGG_PATHWAY	Pyrimidine metabolism	19	0.9	8.30E-02
KEGG_PATHWAY	Amino sugar and nucleotide sugar metabolism	10	0.5	9.40E-02
KEGG_PATHWAY	Mismatch repair	6	0.3	9.60E-02
KEGG_PATHWAY	Propanoate metabolism	8	0.4	9.90E-02
B. Layers				
KEGG_PATHWAY	Focal adhesion	27	2.2	3.40E-03
KEGG_PATHWAY	Lysine degradation	8	0.7	2.40E-02
KEGG_PATHWAY	Propanoate metabolism	6	0.5	6.40E-02
KEGG_PATHWAY	DNA replication	6	0.5	7.40E-02
KEGG_PATHWAY	Amino sugar and nucleotide sugar metabolism	7	0.6	7.80E-02
KEGG_PATHWAY	Apoptosis	11	0.9	9.20E-02

Similar DAVID analyses were performed on the DE gene list obtained from broiler and layer samples. Based on functional annotation, 55 and 34 clusters were obtained in the broiler and layer samples from an initial input of 528 and 430 gene IDs, respectively. With the enrichment cutoff set to >1.0, there were 15 and 10 clusters in the broiler and layer samples, respectively (Additional file 1: Tables S16 and S17). Of these clusters, 2 clusters were enriched and showed exact matches between the broilers and layers (cluster 15 in broilers and cluster 5 in layers). At the pathway level for DE genes, 7 and 9 pathways were identified in broilers and layers, respectively (Table 6). Two common pathways were found, which were Toll-like receptor (TLR) signaling and JAK/STAT signaling.

**Table 6:** DAVID analysis of differentially expressed genes between uninfected and MDV-infection (P<0.05) in broilers and layers

**Category**	**Term**	**Count**	**%**	**P-Value**
A. Broilers				
KEGG_PATHWAY	Cytokine-cytokine receptor interaction	15	2.8	4.90E-04
KEGG_PATHWAY	Notch signaling pathway	7	1.3	4.20E-03
KEGG_PATHWAY	Toll-like receptor signaling pathway	8	1.5	2.10E-02
KEGG_PATHWAY	Jak-STAT signaling pathway	9	1.7	5.30E-02
KEGG_PATHWAY	Cell adhesion molecules (CAMs)	8	1.5	5.80E-02
KEGG_PATHWAY	Focal adhesion	11	2	9.30E-02
KEGG_PATHWAY	Arachidonic acid metabolism	4	0.7	9.80E-02
B. Layers				
KEGG_PATHWAY	Steroid hormone biosynthesis	6	1.4	8.80E-04
KEGG_PATHWAY	Calcium signaling pathway	11	2.5	7.30E-03
KEGG_PATHWAY	Lysosome	8	1.9	1.50E-02
KEGG_PATHWAY	Toll-like receptor signaling pathway	7	1.6	2.10E-02
KEGG_PATHWAY	Vascular smooth muscle contraction	7	1.6	3.50E-02
KEGG_PATHWAY	Drug metabolism	4	0.9	4.20E-02
KEGG_PATHWAY	Metabolism of xenobiotics by cytochrome P450	4	0.9	4.20E-02
KEGG_PATHWAY	Jak-STAT signaling pathway	8	1.9	4.20E-02
KEGG_PATHWAY	Glutathione metabolism	4	0.9	7.00E-02

## Discussion

ASE is a powerful and elegant technique that separates gene expression signals into allelic components resulting in significantly increased sensitivity and added power, and can be used to understand the genetics of gene regulation. In this study, we used ASE to investigate transcriptional regulation in response to MDV infection in two different chicken types, specifically broilers and layers. The primary objective of this study was to identify SNPs exhibiting ASE responding to MDV infection, which would provide a strong foundation for future experiments designed to identify genes conferring genetic resistance to MD. Since it takes 7 days post infection (dpi) for MDV to undergo its first lytic cycle, transcriptional changes are most likely to occur prior to the prevalence of this phenotypic observation and, thus, 4 dpi was chosen as the sampling window captures to capture these transcriptional changes in the birds. In this experiment, we were able to successfully align RNA-Seq reads to the reference chicken genome and identify high quality SNPs in both broiler and layer chickens using our pipeline as described. As a result, we identified 6,132 and 4,528 SNPs significant SNPs in 4,768 and 3,718 genes from broiler and layer samples, respectively.

In chicken, the power of RNA-Seq will not be fully realized until the reference genome sequence is finished [21] (9 of the 39 chromosomes are still wholly absent) and gene models and annotation become more complete. For example, while only exons should be transcribed, nonetheless, a large percentage of the SNPs, when classified using ANNOVAR, fell into categories such as downstream, intronic and intergenic regions. Poor genome annotation is most likely responsible for the classification of SNPs in non-exonic regions as we obtained high quality reads with excellent coverage. We are in the process of developing new gene models based on our experimental RNA-Seq data from this study and additional ones.

At the SNP and gene level, 72 SNPs and 71 genes (Table 2) were common between ASE broiler and layer samples. The occurrence of identical SNP positions was negligible, which was expected given the wide genetic divergence between these two chicken types, and therefore is due to chance. Comparison of the enriched pathways obtained from ASE SNP positions also showed little similarities between the two bird types (Table 5). We conclude, based on the lack of concordance between the lists of identified SNPs, genes and pathways, there is no evidence to suggest common genetic elements that respond to MDV infection in these two bird types; this result also supports the notion that MD genetic resistance is complex and can result from different gene combinations. If true on a broader scale, then genetic markers for complex traits such as genetic resistance to MD based on ASE screens will need to be performed on each population and cannot be extrapolated from one bird line to another.

The chicken major histocompatibility complex (MHC) plays an important role in the determination of resistance to MDV [13]. The chicken chromosome 16 contains 3 loci namely, the B locus, the Y (or Rfp-Y) locus and the nucleolar-organizing region (NOR). Loci B and Y are unlinked and evolve independently [22]. In this study, in our analysis of nonsynonymous ASE SNPs in broilers and layers, we found a SNP associated with a gene that encodes for class I alpha chain of the Rfp-Y loci (ENSGALG00000024348, Additional file 1: Table S15). This result supports previous findings that have linked MHC to MDV resistance. However, our predications are limited due to incomplete sequence information of chromosome 16 for both galGal3 and galGal4 chicken genome builds. This limitation combined with the fact that many of the relevant genes have multiple family members hindered our effects to ascribe ASE on MHC genes and the pathways downstream.

Despite hindrances such as incomplete gene models, we successfully identified two pathways namely, TLR signaling and JAK/STAT signaling [23], by analyzing DE in broilers and layers. Recently, Smith and co-workers [24] reported the enrichment of the above-mentioned pathways in layers in response to MDV infection. Our data suggest that broilers also show similar pathway enrichment, thus, we conclude they have similar pathways for responding to MDV infection.

Understanding the biological implications of large datasets generated by RNA-Seq can be challenging. Moreover, to identify genetic effects that play an important role in downstream regulation and expression is a major concern in fully understanding the significance of pathways or networks. One hypothesis proposed by Clark et al. [25] was to consider the association of upstream gene(s) or loci as genetic factors that influence downstream processes such as gene expression. In other words, downstream DE in genes could be controlled by genetic factors (genes) upstream in key regulatory pathways. To dissect the influence of ASE on downstream DE analysis, we searched for possible associations between pathways enriched for genes with differential expression (i.e., DE gene lists) and genes exhibiting variation in transcriptional response (i.e., ASE gene lists).

**Figure 1:**
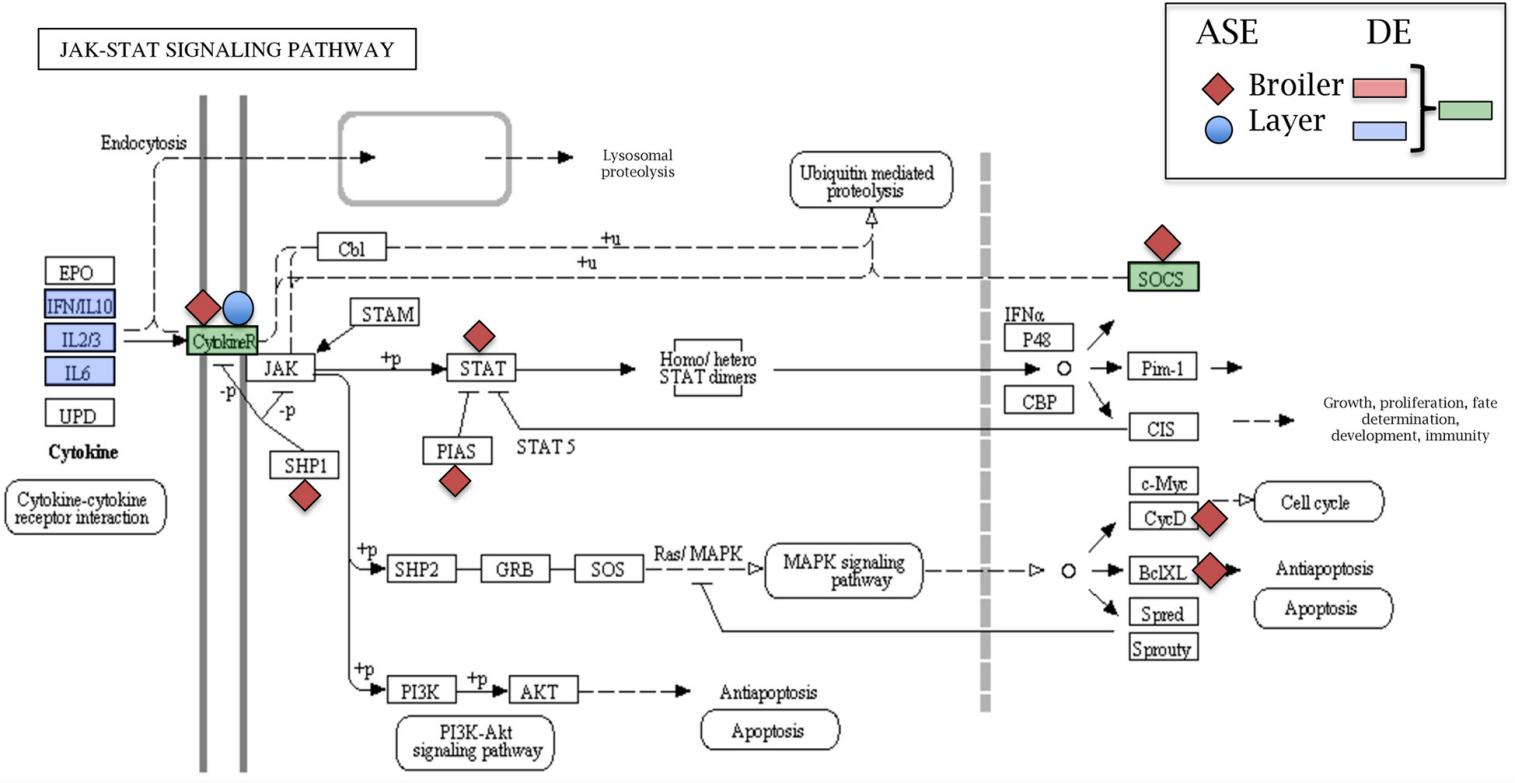
**The JAK/STAT signaling pathway in response to MDV infection.** The JAK/STAT signaling pathway is displayed and genes that exhibit either differential expression (DE) or allele-specific expression (ASE) following Marek’s disease virus (MDV) infection in both broilers and layers are shown.

**Figure 2:**
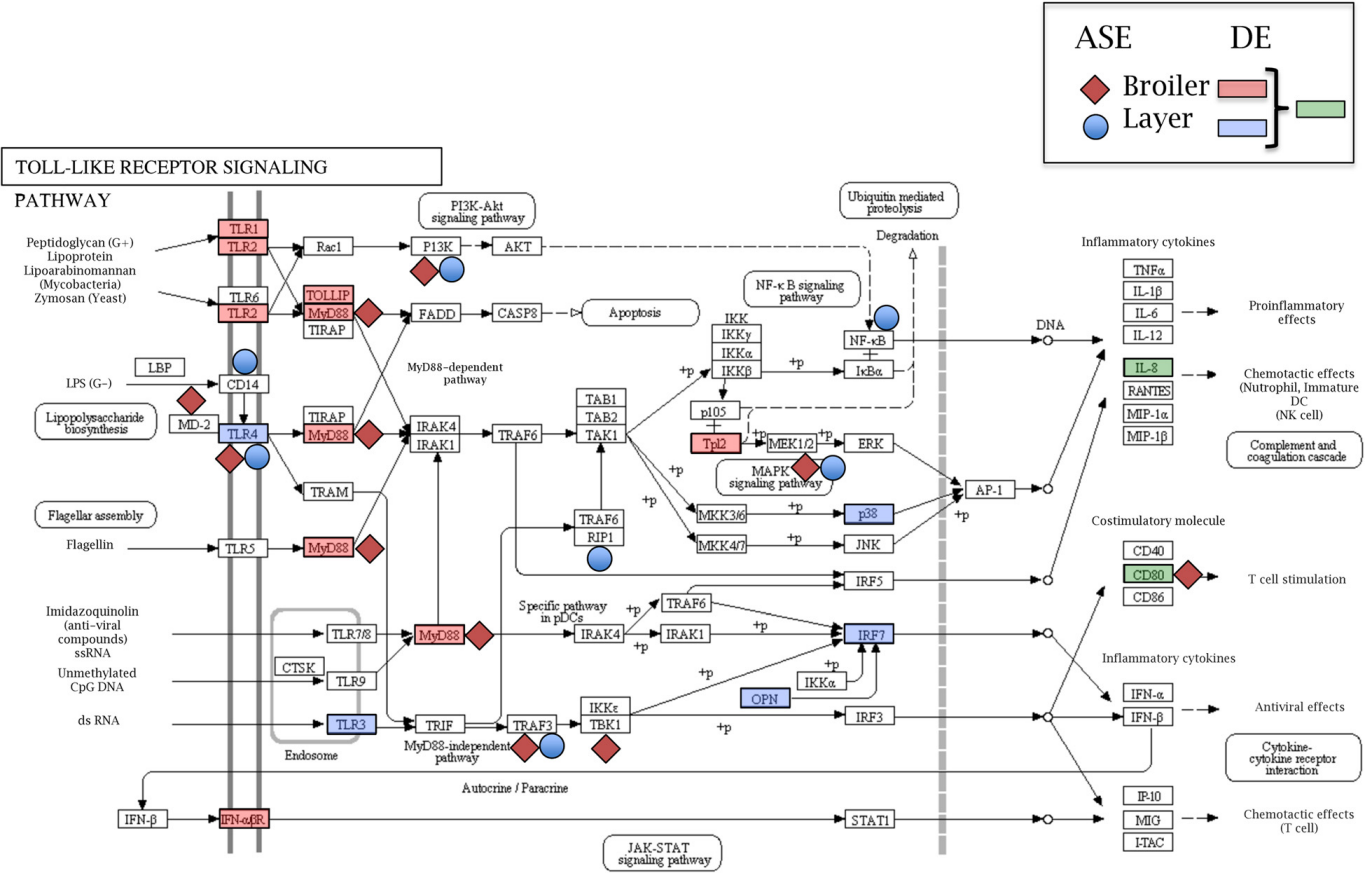
**Toll-like receptor signaling pathway in response to MDV infection.** The Toll-like receptor signaling pathway is displayed and genes that exhibit either differential expression (DE) or allele-specific expression (ASE) following Marek’s disease virus (MDV) infection in both broilers and layers are shown.

Our data supports the above hypothesis of an underlying genetic component amplifying the expression of downstream genes in a pathway. Specifically, examination of the two common pathways (TLR signaling and JAK/STAT signaling), as shown in Figures 1 and 2, genes exhibiting ASE in response to MDV were found at the beginning of each pathway. Specifically, genes encoding CD14, LY96 (aka MD-2), TLR4, and MyD88 exhibit ASE in response to MDV infection and are found at the start of the TLR receptor-signaling pathway, which contains genes with DE in layers and especially for broilers. Likewise, IL5R, IL21R of the Cytokine R module, PTPN6 (aka SHP1), and PTPN11 (aka SHP2) are all found at the beginning of the JAK/STAT pathway for broilers and layers. It is also interesting to note that growth hormone (*GH*), one of the three MD resistance genes identified [8], also lies at the start of the JAK/STAT pathway though we could not evaluate for ASE due to the lack of SNPs in the F_1_ progeny. Interestingly, nuclear factor-kappa B (NF-κ B), which regulates genes associated with cell survival, proliferation, programmed cell death (PCD), stress, inflammation, and immunity was previously shown to be a key component of MDV infection [26] and showed ASE in the layers in this study as well as being a member of the TLR signaling pathway among others (e.g., viral carcinogenesis, B and T cell receptor signaling, chemokine signaling). If the genes at the beginning of the aforementioned pathways do confer genetic resistance to MD, then this has strong implications on how to analyze RNA-Seq data to identify strong candidate genes in other complex traits.

## Conclusions

In conclusion, we have successfully identified SNPs associated with ASE in both broilers and layers chicken types, as well as revealed genes and pathways that were common or unique to each bird or analysis type. Thus, the method is powerful for identifying SNPs and genes that exhibit differential allelic response to MDV infection as well as providing candidate markers and genes for MD genetic resistance. Furthermore, we believe that greater exploration should be performed placed on genes exhibiting ASE at the beginning of pathways identified by gene expression enrichment analysis. The larger question that still remains is how much do genes exhibiting ASE (i.e., expression variation) account for overall genetic variation in complex traits such as MD genetic resistance? To answer this question, we have developed a custom chip containing ASE SNPs that is being genotyped on an MD resource population, which will determine, if present, the size of effect and which alleles are favorable for genetic resistance to MD.

## Methods

### Animals and experimental design

Two chicken types, commercial broilers (meat-type) and experimental layers (egg-type), were used in this study. The broiler chickens were either chicks from two outbred Cobb-Vantress pure lines or their F_1_ progeny as determined by the experiment. The layer chickens were F_1_ progeny from intermating Avian Disease and Oncology Laboratory (ADOL) lines 6_3_ and 7_2_, two highly inbred layers lines that are MD resistant and susceptible, respectively.

MD incidence in the broilers was measured in two trials. For each trial, ~100 day old chicks were inoculated intra-abdominally with 2,000 pfu MDV (686 strain). All the chickens were housed in Horsfall-Bauer isolators during the entire experiment, and mortality and MD incidences were measured daily or at the termination date (8 weeks post inoculation) by necropsy.

To generate material for determining ASE, F_1_ progeny broiler and layer chicks were randomly divided into either treatment group (challenged) or control group (unchallenged). For the viral challenge, 2,000 pfu MDV (686 or Md5 strain for broilers and layers, respectively) was injected intra-abdominally at 2 days of age. At 4 days post infection (dpi), 7 birds from each group were euthanized, and splenic tissue samples collected and stored in RNAlater **®** (Ambion, Austin, TX, USA). Animal care and management followed the ADOL animal care and usage committee policy.

### RNA extraction and sequencing

Total RNA was extracted from splenic tissues using the Stratagene Absolutely RNA Miniprep kit (Santa Clara, CA, USA). RNA quality was determined using an Aligent Bioanalyzer 2100 lab-on-a-chip and only samples with RIN scores over 7 were submitted for sequencing.

Sequencing was done using Illumina sequencing platforms. Broiler RNA (single end, 75 base read lengths) was sequenced at Michigan State University Research Technology Support Facility (http://www.rtsf.msu.edu) using an Illumina Genome Analyzer II. Layer RNA (pair-end, 100 base read lengths) was sequenced at DNA Landmarks (Montreal, Canada) using an Illumina HiSeq.

### Mapping and assembling

All RNA-Seq data reads were trimmed of sequencing adapters, and sequence statistics performed using FASTQC program [27]. Based on these results, the reads were further trimmed using Sickle, a program (https://github.com/najoshi/sickle) that removes reads containing ‘N’ as well as pairing reads, if paired end sequencing was used for down stream alignment. A minimum post-trimmed length of 50 bases was set irrespective of single or paired-end reads. The resulting FASTQ files were processed by Tophat (Version 2.0.4) [28]. Alignments were invoked inside Tophat using Bowtie (Version 2.0.6; [14]) by providing the library insert size for paired ended reads, the reference chicken genome (galGal3), and the ENSEMBL chicken gene GTF file list (Version 67). Read quality statistics of the resulting alignment files were analyzed using SAMStat [29] and the files were used to call SNPs and estimate expression of genes.

### SNP detection, ASE estimation and functional annotation of SNPs

Unique read groups were added to each alignment file and population level SNPs within each group (broiler or layer) were called using Freebayes (Version 0.9.6), a Bayesian SNP calling program [15]. The variant calling format (VCF) files [30] derived from infected and uninfected birds were merged with VCFtools [30] and further filtered based on quality scores. SNP call statistics subsequently determined by VCFtools and the raw SNPs were further filtered on the basis of quality (Q=100), thereby, additionally increasing the stringency of called SNPs. Subsequently, a 4-column file with chromosome number, nucleotide position, number of reference SNPs, and number of alternate SNPs were parsed out from each sample from the VCF file. Data was formatted and read into SAS for ASE estimation.

The frequency of each SNP allele expressed as a proportion of total number of reads for the allele present in the reference genome, was analyzed using the following linear model:

$$Y_{\mathrm{ijk}=\mu +T_{i}+C_{j}+TC_{\mathrm{ij}}+\varepsilon_{\left(\mathrm{ij}\right)k}}$$

Where (*Y*_*ijk*_) is the allele frequency in the k^th^ individual (k=1,6) within the i^th^ treatment and j^th^ cross, T_i_ is the effect of treatment, C_j_ is the effects of cross, and TC is the interaction of treatment with cross. The dependent variable was the reference allele frequency with variation among biological replicates within treatment used for testing. SNPs were eliminated for a bird type if they had less than 2 samples for any treatment group. A pooled error term was used to test for significance. As a first pass, all genes passing the 0.05 level of significance where chosen for further analysis using pathway enrichment, in this way the rate of Type 2 errors was reduced, yet allowed for a final test using Fisher exact test for pathway enrichment which controlled for Type 1 errors.

ANNOVAR [17] was used to functionally annotate the putative SNPs. The ANNOVAR database was set up as described [17] and each SNP was classified based on its position in the reference chicken genome as exonic, intronic, intergenic, 5’ UTR, 3’ UTR, splice acceptor or donor site, downstream or upstream. Functional annotation such as nonsynonymous, synonymous, stop codon gain or loss, and amino acid changes was also determined for each SNP.

### Differential expression (DE) analysis

To test DE, alignments were analyzed using DESeq, a negative binomial distribution-based [20] approach within R [31]. For input data, the number of reads (count data) that uniquely mapped to an exon was counted using Htseq-count [18] and the provided reference chicken gene list. The reads were processed in Htseq using a **‘**union**’** overlapping mode, **‘**gene**’** as feature, and with no strand-specificity. The resulting data from all the individual samples were converted to text file imported into R and analyzed. A portion of the reads (5.7 and 12.6 million reads on average in broiler and layer samples, respectively), were assigned to “no feature.”

### Pathway analysis using DAVID

ASE and DE significant genes (p<0.05) lists were submitted to DAVID [16]. The analysis classification stringency was set to the highest level with suitable controls. The resulting clustering was then limited to an enrichment score of >1.00 and FDR for multiple testing was performed by the Benjamin and Hochberg method invoked within DAVID. Kyoto Encyclopedia of Genes and Genomes (KEGG) pathway analysis was also performed using DAVID.

## Competing interests

The authors declare that they have no competing interests.

## Authors’ contributions

SP conducting all the bird experiments, prepared all the RNAs, conducted all of the bioinformatic analyses, and prepared the manuscript. WMM assisted in the bioinformatic analyses, performed all the statistical analyses, and reviewed the manuscript. ABP assisted in the bioinformatic analyses, and reviewed the manuscript. RO provided the Cobb-Vantress broilers and reviewed the manuscript. HHC conceived and coordinated the study, and helped write the manuscript. All authors read and approved the final manuscript.

## Supplementary Material

Additional file 1 **Table S1.**Broiler raw reads, alignment, and raw SNPs. **Table S2.** Layer raw reads, alignment, and raw SNPs. **Table S3.** List of ASE SNPs in broiler samples (p<0.05) in response to MDV infection. **Table S4.** List of ASE SNPs in layer samples (p<0.05) in response to MDV infection. **Table S5.** Common SNPs between broilers and layer ASE samples in response to MDV infection. **Table S6.** ANNOVAR classification of broiler SNPs based on function. **Table S7.** ANNOVAR classification of layer SNPs based on function. **Table S8.** Genes differentially expressed (P<0.05) following MDV infection in broilers. **Table S9.** Genes differentially expressed (P<0.05) following MDV infection in layers. **Table S10.** Functional annotatiion clustering of ASE genes in response to MDV in broilers using DAVID. **Table S11.** Functional annotatiion clustering of ASE genes in response to MDV infection in layers using DAVID. **Table S12.** Functional annotatiion clustering of nonsynonymous genes in response to MDV infection in broilers using DAVID. **Table S13.** Functional annotatiion clustering of nonsynonymous genes in response to MDV infection in layers using DAVID. **Table S14.** Common nonsynonymous genes between broilers and layers. **Table S15.** Functional annotation of 14 common nonsynonymous genes between broilers and layers. **Table S16.** Functional annotatiion clustering of DE genes in response to MDV infection in broilers using DAVID. **Table S17.** Functional annotatiion clustering of DE genes in response to MDV infection in layers using DAVID. Click here for file

Additional file 2 **Figure S1**Distribution of SNPs exhibiting ASE in response to MDV infection in chicken chromosomes. Click here for file
