# Supplementary material for: Comparison and contrast of genes and biological pathways responding to Marek’s disease virus infection using allele-specific expression and differential expression in broiler and layer chickens
Source: BMC Genomics. 2013 Jan 30;14:64. doi: 10.1186/1471-2164-14-64 (PMC3599046; doi:10.1186/1471-2164-14-64)

Supplemental Figure 1. Distribution of SNPs exhibiting ASE in response to MDV infection in chicken chromosomes.


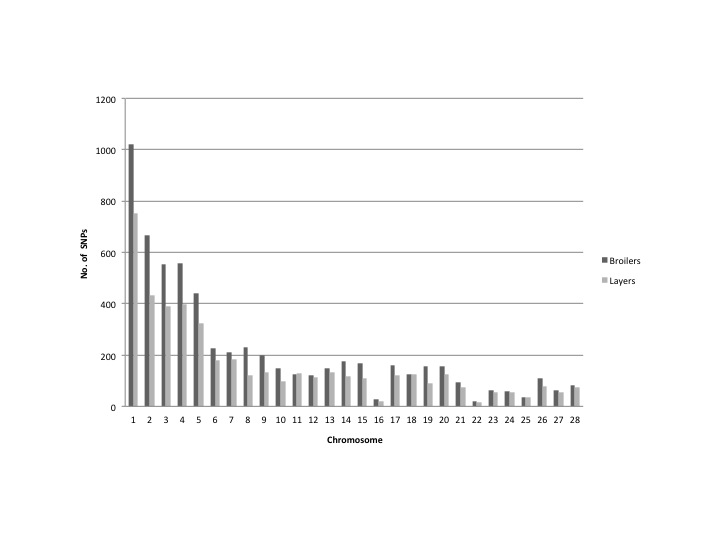

Supplement: Additional file 2 — Figure S1Distribution of SNPs exhibiting ASE in response to MDV infection in chicken chromosomes. [file 1471-2164-14-64-S2.docx]
